# What factors influence recruitment to a birth cohort of infants with Down’s syndrome?

**DOI:** 10.1136/archdischild-2017-314312

**Published:** 2018-03-08

**Authors:** Georgina M Williams, Patricia Neville, Kathleen M Gillespie, Sam D Leary, Julian P Hamilton-Shield, Aidan J Searle

**Affiliations:** 1 The NIHR Bristol Biomedical Research Centre, Nutrition Theme, University of Bristol, Bristol, UK; 2 Diabetes and Metabolism, Translational Sciences, University of Bristol, Bristol, UK; 3 Bristol Dental School, Faculty of Health Sciences, University of Bristol, Bristol, UK

**Keywords:** down’s syndrome, recruitment, qualitative research, comm child health, birth cohorts

## Abstract

**Objective:**

To understand how to maximise recruitment of young infants with Down’s syndrome (DS) into research through qualitative interviews with parents and care providers. In complex neonatal and genetic conditions such as DS, frequently diagnosed after birth, parents may go through a period of adaptation. These factors need consideration when overcoming barriers to recruitment.

**Participants and design:**

Participants, who were drawn from health professionals and volunteers working with families experiencing DS, were recruited using a purposive sampling strategy. Semistructured telephone interviews were completed with nine paediatricians, three research nurses and six family support workers. Five of those interviewed had a child with DS. The interviews were transcribed and analysed thematically.

**Results:**

A positive decision to take part in a ‘from-birth’ cohort study depends on factors such as the child’s overall health, parent demographics (educational background and ethnicity), medical interactions that take place with the families (communication) and study logistics. The data suggest that recruitment methods need to take all these factors into consideration. Multiple recruitment methods should be considered including face to face, through parent and support groups, websites and social media. There also needs to be flexibility in the research timings to fit around the needs of the child and parents.

**Conclusion:**

Researchers need to be aware of the variable responses elicited by families to a diagnosis of DS for their baby and be sensitive to the child’s current medical status. This does not preclude recruitment into studies, but to maximise uptake good communication and flexibility is essential.

**What is already known on this topic?**

- Parents who have a baby with Down’s syndrome often experience a period of adjustment to the diagnosis.
- Recruiting children with Down’s syndrome into research studies can be challenging.

**What this study adds?**

- Multiple recruitment strategies including the use of social media attracts families into research for infants with Down’s syndrome.
- Allowing flexibility into recruitment protocols can improve participation.

## Introduction

Birth cohorts targeting Down’s syndrome (DS) or other genetic conditions are rare. However, prospective cohort studies are essential for understanding the natural history of these conditions.

Recruiting any family with a newborn into a research study is challenging, but the potential ‘setback’ of a diagnosis of DS compounds the difficulty. The majority of diagnoses are made at birth or shortly afterwards^1^: around 92% of prenatal diagnoses lead to a termination.^1^ Babies with DS have associated conditions often diagnosed neonatally including congenital heart disease, haematological and gastrointestinal problems.^2^ These may have a significant impact on the baby, family and early bonding opportunities. Studies exploring how parents cope and adapt to receiving the diagnosis of DS have described the increased stress compared with parents of a typical newborn.^3^ Some families will respond with shock and upset, while others will positively thrive.^3 4^

The Feeding and Autoimmunity in Down’s Syndrome Evaluation Study (FADES) is a birth cohort of infants with DS exploring links between early life events and autoimmunity. The present qualitative study explored ways to improve recruitment during the feasibility phase and to inform future birth cohorts recruiting chromosomal/genetic anomalies or complex neonatal conditions.

## Methods

### Participants

Paediatricians, research nurses (RNs) and family support workers were recruited following a purposive sampling strategy. Selection was made for experience in supporting and caring for infants with DS and their families, during the months after birth, or recruiting new parents to research studies. This study was advertised among members of the Down’s Syndrome Medical Interest Group. Family support workers (FSWs) and charity workers from the Down’s Syndrome Association and Down’s Syndrome Scotland were approached via contacts already known to the FADES team. FADES local collaborators, community paediatricians (CPs), neonatologists and RNs were also invited to take part.

Of the 18 interviewees, two-thirds were clinical (nine paediatricians and three RNs) and one-third were FSW. Of those interviewed, five had a child with DS.

### Interviews

A topic guide for the interviews was developed following a literature review of parental adjustment to the diagnosis of DS and recruitment issues for neonatal studies. The guide included items that explored the interviewee’s experience of working with families having a child with DS and their views on recruiting this group into research studies. In keeping with the iterative nature of qualitative methods, the topic guide was revised following initial interviews. GMW conducted the semistructured telephone interviews with the participants. Recruitment ended when the interviews were no longer revealing any new information, and data saturation had been reached. The interviews were between 25 min and 1 hour in duration.

### Analysis

Interviews were digitally audio-recorded and transcribed verbatim by a university-approved transcription service. Thematic analysis was then undertaken following the guidelines stipulated by Braun *et al*.^5^ Thematic analysis is described as a flexible and useful research tool that can potentially provide a rich and detailed yet complex account of data.^5^

The analytical process undertaken is summarised in box 1.

Box 1 **Process undertaken for the thematic analysis**

1. Familiarisation of dataset: transcripts were read, re-read and discussed.
2. Defining a coding framework: a subsample of transcripts were initially open coded; disparities in codes were discussed until consensus on codes was achieved. During coding of the dataset, further revisions were made before a definitive coding framework was established.
3. Coding: all transcripts were coded in Nvivo V.10.
4. Themes: coded transcripts were reviewed to identify the emerging themes from the dataset.

## Results

Thematic analysis led to the identification of three key themes: (1) ‘Family context’, (2) ‘Interactions’ and (3) ‘Research factors’.

1. ‘Family context’ included the subthemes: 1.1: ‘Family demographics’, 1.2: ‘Family attitudes and values’ and 1.3: ‘Child health status’.
2. ‘Interactions’ included the following subthemes: 2.1: ‘Communication with parents’ and 2.2: ‘Recruitment approach’.
3. ‘Research Factors’ included the following subthemes: 3.1: ‘Burden’, 3.2: ‘Gatekeeping’ and 3.3: ‘Logistics’.

### Family context

#### Family demographics

The educational background, socioeconomic status, ethnicity and the beliefs of families were described by health professionals to be potential barriers to engaging in research.

… I think the more educated parents are more likely to consider it. I think a lot of the families that I work with come from cultures and communities where participation in research is almost unknown. (CP)

Certainly, we have some families who live in incredibly deprived circumstances and life being what it is these are also often the ones who may not be English first language speakers, so they may have difficulties. (CP)

### Family attitudes and values

Health professionals suggested that some parents are more likely to consent to a study given their ‘natural stance’ and values.

Having a fairly altruistic view of what’s happening… a mum being diagnosed with a Down’s syndrome baby at 20 weeks is a bit of a shock; to be recruited at that time, and agree, and wasn’t going to have that baby terminated. Yes, that type of stance gets selective, doesn’t it? But that sort of a mum; a mum that says, ‘This baby has Down’s syndrome. so, what?’. (RN who also runs a support group)

Altruism featured as a big motivator for parents in taking part in research:

I think a lot of families just want to help, you know, they want to help any families that may be coming up in the future, and again it’s usually the more positive people that want to do that, and the confident ones I suppose. (FSW who runs a support group)

There was a degree of concern about exacerbating any anxieties for families by inviting them to participate in research.

I think maybe they might be concerned that you might be asking questions that might unearth some things that they’re not ready to address. (RN)

One health professional described parents falling into one of two groups: those that are either very involved or not psychologically prepared.

Well in a way you have got more extremes there because you will have those, ‘I have got a child with a disability. I want to do everything I can for them or for others’ and they are more likely to agree. Equally, ‘This is a shock to me. I am not used to this. I don’t know what I am doing. There is so much to learn. There is so much to get my head around. I can’t take that on board as well’. (RN)

### Child health status

A significant factor for parents considering participation in a research study is their child’s health. Around 50% of infants with DS have congenital heart disease,^2^ potentially life threatening and requiring surgery in the neonatal period, which becomes a parental and clinical priority. Clinicians emphasised that although an important consideration, it should not preclude research engagement.

With 54% of kids being born with some kind of cardiac condition, they may be the ones that are still in hospital further down the line, or kids who may have some kind of gut malformation will still be in the hospital for longer. For those parents, it may be that they haven’t really come to terms or thought about the Down’s syndrome. It’s really more the getting the health issues seen to. (FSW)

### Interactions

#### Communicating with parents

Health professionals emphasised the importance of effective communication when sharing the diagnosis of DS and recruiting families. Engaging parents in a study requires the involvement of someone with an established rapport with that family. The interviewees identified doctors, allied healthcare professionals, support workers and other parents.

So, through the Down’s Syndrome Association, local parent groups, and making them aware. Then, of course, people who have regular contact with the families…if you’ve got somebody who the parent trusts introducing the idea of the research, they’re more likely to be receptive to it, and think, Yes, that sounds like a genuine thing that we should consider. (CP)

Poor communication around the time of diagnosis and when discussing the outcomes for children with DS makes a lasting impression on families. This may affect future relations with medical professionals and researchers.

I mean if you’ve had a bad experience, and the whole thing about being told about your child’s diagnosis, well it’s always going to be a difficult memory, I would imagine, but I do think whether or not people are going to want to engage with their clinicians in research has got to be affected by the way that their relationship started off. I’m sure it’s related. (FSW and mother)

Explanations for inadequate communication with parents were: lack of time, inappropriate timing, language barriers and the limited ability of some families to read and understand participant information sheets.

[Y]ou’re so busy and you’re trying to get things right, but actually you say the wrong things because of your busyness. You know that these parents are going to remember exactly what you say years later… (FSW, mother and health visitor)

[T]he length and the complexity makes probably the biggest difference and also we never actually check how well parents can read …I think sometimes their ability to read and understand what they’ve got to do is sometimes a challenge for some parents. (Paediatrician/neonatologist)

#### Recruitment approach

Interviewees discussed different recruitment methods, most preferring face to face but also acknowledging the roles of social media, websites and invitation letters. Interviewees regarded the communication network between families (local parent baby groups and DS specific social media groups) as an important way to disseminate information regarding research.

But if you had a paediatrician that you didn’t feel was specifically interested and engaged with your child, particularly one of those who talks over the child like they don’t exist or something then you are probably not going to be engaged with it…Whereas you might actually take it on board from a support group, your local Down’s Syndrome group, other parents, even social media. (FSW and mother)

When a family might be most receptive to being approached for research recruitment varies. Each family has their own experience and response to having a new baby with DS. Participants suggested that greater awareness of timelines might help researchers interact with the families.

For example, families who received an antenatal diagnosis of DS deciding to continue with the pregnancy may be more receptive to participating in research early.

I’ve found that we’ve got one or two families that have had pre-diagnosis, they’ve known they were going to have a child with Down’s Syndrome, and they’re very keen to help us, and do, whereas probably families who’ve found it a shock take more getting used to it. Whereas some of the ones that have… They’re already pre-prepared, and they’ve looked at the research…. (FSW and mother)

The importance of parental bonding with their new baby and recovering from birth means that many felt there should be a period before introducing the idea of a study.

I suspect that you’ve also got to ensure that all that normal bonding stuff is happening as well, and that the whole talking to people about being a part of a research study when they’re still getting their heads around questions like, ‘Can I love this child?’ and, ‘Is this child going to be part of our family?’. (FSW and parent)

The timing of recruitment will depend on aspects of the diagnosis. One CP referred to parents who were unwilling to engage with them until the results of the karyotype had been given.

Some families won’t agree to have an echocardiogram until they have got the blood test result back because we might have made a mistake and the baby wouldn’t need it. I think with some you won’t really be able to get any further until you have got that result back. (CP)

### Research factors

#### Burden

For any parent with a new baby, life can be chaotic. Thus, involving them in research requires the design to fit around other demands. Interviewees talked about the increased challenges which parents of a DS baby may have and how research might accommodate these.

Well it is the time – both the time taken to do it but also the timeframe and how it fits around where they are … if it is invasive tests or something that having the flexibility to fit that around what they are already doing…sometimes it is things like childcare for other children because they are quite happy to go along with the child with Down’s Syndrome and participate in something. But if it is supported- (FSW)

Some health professionals felt that being part of a research study may be a supportive experience helping parents at a difficult time.

Sometimes they probably just need someone who is there special for them that they can talk to who if they can’t find an answer they can go off and find an answer for them and come back to them …That is where the research nurse can come in and do the extra. ‘You are special to me and I am going to help you’. (RN)

#### Gatekeeping

Before eligible families are even approached, there were several barriers highlighted by the interviewees. Institutional approvals are required, and once approved, ‘recruiters’ place restrictions on whom they will target either consciously or subconsciously. To be able to contact all potential families and minimise gatekeeping both, health services and voluntary organisations/charities should be used for recruitment:

I do think there is something to be said about a sort of two-pronged approach. … within the hospitals and SCBU staff, there is this kind of gate-keeping thing going on. … people have to find us via the DSA website; despite the fact we’ve left bits and bobs…. (FSW and parent)

#### Logistics

Opportunities to interact with families and recruit into studies may be infrequent. Some babies will have a period in a neonatal unit, and this was described as a good time to introduce research. Those with cardiac and bowel problems may have multiple appointments, but this was perceived as a more unsettled time when parents may not be able to think about enrolling into a study.

They may be in the special care unit for an extended period of time in which case the neonatal consultant would be, you know, most suitable to do that. (CP)

So, I think they are probably less likely to engage until they have got through that surgery bit. (FSW and mother)

If a baby with DS has no additional medical issues, the CP follow-up appointments may vary. Most adhere to current guidelines: following up at 3 and 6 months, then annually until 5 years.

The problem you will have is that everywhere does it differently… You have to find out, in each area, how they manage children with Down’s syndrome and make sure your message is getting to the right people. (CP)

Finally, there was some consensus that 3 months may be a good age to approach families. Parents were described as having ‘got over’ the newborn period and adjusted or adapted to the diagnosis of DS.

If you don’t get them in the new-born period then I think there is a sort of sense in which perhaps you wait until they come back to hospital for the first visit. In our case it would be at three months. (CP)

## Discussion

This qualitative study aimed to understand factors involved in recruiting new families to birth cohort studies of babies with DS to optimise recruitment to the FADES study. The findings could also be used as a paradigm for recruiting neonates with other complex conditions.

The main findings suggest that successful recruitment requires a variety of approaches. Although parents often have a good relationship with their medical team and like being recruited by this traditional route, trust can be marred by difficult experiences at diagnosis and poor communication. Health professionals may act as gatekeepers and alternative routes may circumvent this. Using social media, websites and parent groups were suggested as alternative trusted sources. Understanding the dynamics around the time of diagnosis and following months helps in planning study logistics. Making the timings of recruitment and data collection flexible, particularly when babies have other complications, is important. Interviewees did not discuss how patient and public involvement (PPI) could inform this type of research; however, PPI advice was sought for FADES.

This study has benefitted from the variety of opinions gained, particularly those of affected parents. All interviewees were candid in their responses. The FSWs receive training in family support, but this is unlikely to include research recruitment. Thus, the responses they gave in relation to research likely represent their own experiences and opinions. The paediatricians were all members of the Down’s Syndrome Medical Interest Group or involved in FADES that may have caused bias. However, this group has a wealth of experience with DS families and research.

Interviews were conducted by telephone rather than face to face, enabling participation from a geographically wide area. Although occasionally viewed as inferior to face-to-face interviews, Sturges and Hanrahan^6^ found no significant differences and suggested there may be notable benefits, particularly allowing participants a degree of anonymity.

Previously published research involving infants with DS is limited, and details regarding recruitment are lacking. An Health Technology Assessment study exploring the feasibility of recruiting children aged 1–11 years with DS into an otitis media, treatment study, interviewed clinicians and parents regarding recruitment.^7^ Their conclusions were similar: any research needs to account for both the shared experiences of parents with a baby with DS and the variety of personal experiences. However, alternative recruitment strategies were not explored, and more novel recruitment methods may allow some of the variation in personal perspectives and timings to be navigated.

## Conclusion

This qualitative study provides insight into the issues surrounding recruitment of babies with DS. Recruitment should include the use of clinicians and alternative methods including social media, parent groups, charities and websites. From the knowledge provided by those interviewed, families will have different experiences in the first few months of their child’s life depending on whether they had an antenatal diagnosis, how they adapt and adjust to the diagnosis, related medical conditions and the support they receive. This information helped develop the FADES protocol, which has the flexibility to allow families and their babies to fully participate dependent on their own individual and medical circumstances.
